# Propofol for depression: mechanisms and therapeutic potential

**DOI:** 10.3389/fphys.2026.1876988

**Published:** 2026-08-10

**Authors:** Dan Yu, Ye Liao, Jie Li, Aiqiu Zheng, Xin Meng, Shumin Gong, Can Li, Kunwei Wu

**Affiliations:** 1 Jiangsu Province Key Laboratory of Anesthesiology and Brain Science, NMPA Key Laboratory for Research and Evaluation of Narcotic and Psychotropic Drugs, School of Anesthesiology, Xuzhou Medical University, Xuzhou, Jiangsu, China; 2 Department of Pathogen Biology and Immunology, Jiangsu Key Laboratory of Immunity and Metabolism, Jiangsu International Laboratory of Immunity and Metabolism, Xuzhou Medical University, Xuzhou, Jiangsu, China; 3 Department of Anesthesiology, The Affiliated Hospital of Xuzhou Medical University, Xuzhou, Jiangsu, China

**Keywords:** anesthesia, depression, drug repurposing, GABAergic transmission, propofol, rapid antidepressant, treatment-resistant depression

## Abstract

Propofol, one of the most widely used intravenous anesthetics, is undergoing a transformative identity shift from a purely surgical agent to a potential therapeutic tool for neuropsychiatric disorders. This review synthesizes current evidence on the emerging field of propofol therapeutics, with a focus on its rapid antidepressant, anti-anhedonia, and anxiolytic effects. We first examine preliminary clinical evidence from open-label studies suggesting that propofol may produce dose-dependent mood-elevating effects in treatment-resistant depression; however, this evidence remains limited by small sample sizes and lack of large controlled trials. Next, we explore putative mechanisms, including GABA_A_ receptor modulation, dopamine transporter inhibition, BDNF/TrkB signaling, and network-level reorganization of the default mode network and sleep architecture. We then compare propofol with other anesthetic agents possessing antidepressant properties, particularly ketamine, highlighting their distinct receptor targets, efficacy profiles, and safety considerations. Finally, we discuss major challenges including optimal dosing strategies, tolerance with repeated administration, long-term safety concerns, and implementation barriers. Despite these challenges, propofol represents a promising candidate for anesthetic repurposing in psychiatry, offering a mechanistically distinct alternative to ketamine that targets both GABAergic and dopaminergic systems with a well-established clinical safety profile for surgical anesthesia.

## 1 Introduction

Major depressive disorder (MDD) is a leading cause of global disability. According to the World Health Organization and the Global Burden of Disease Study, approximately 280 million people worldwide suffer from depression (Diseases GBD and Injuries C, 2024). The lifetime prevalence of MDD exceeds 15% in high-income countries, and the disease is projected to become the leading contributor to the global burden of disease by 2030 (Malhi and Mann, 2018; Malhi et al., 2026). Despite decades of drug development, the pharmacological treatment of depression remains constrained by two fundamental limitations. First, conventional antidepressants—selective serotonin reuptake inhibitors (SSRIs), serotonin-norepinephrine reuptake inhibitors (SNRIs), and tricyclic antidepressants—require several weeks (sometimes longer) to produce clinically meaningful symptom improvement (Malhi and Mann, 2018; Malhi et al., 2026; Trivedi et al., 2006). This delayed onset creates a dangerous window during which patients remain at elevated risk for suicide and functional deterioration. Second, the efficacy of these agents is limited. The landmark Sequenced Treatment Alternatives to Relieve Depression (STAR*D) trial revealed that only approximately one-third of patients achieve remission after first-line treatment; after four sequential treatment steps, nearly one-third remain symptomatic despite adequate pharmacotherapy (Rush et al., 2006).

The identification of ketamine as a rapid-acting antidepressant marked a paradigm shift in the field. In 2000, Berman and colleagues reported that a single subanesthetic dose of ketamine produced rapid (within hours) and sustained (up to 72 hours) antidepressant effects in patients with treatment-resistant depression (TRD) (Berman et al., 2000). Subsequent studies confirmed and extended these findings (Zarate et al., 2006; Murrough et al., 2013), leading to the regulatory approval of esketamine (the S-enantiomer of ketamine) nasal spray by the U.S. Food and Drug Administration (FDA) in 2019 for TRD and for major depressive disorder with acute suicidal ideation (Kim et al., 2019). The success of ketamine has catalyzed a broader investigation into the repurposing of anesthetic agents for psychiatric indications. Anesthetics—by virtue of their potent and rapid modulation of central nervous system excitability—are uniquely positioned to address the slow onset and incomplete efficacy that characterize conventional antidepressants (Krystal et al., 2019).

Propofol (2,6-diisopropylphenol) is the most widely used intravenous anesthetic in clinical practice. First synthesized in 1977 and introduced into clinical practice in the 1980s, propofol has become the agent of choice for induction and maintenance of general anesthesia (Schraag et al., 2018), sedation in intensive care units, and procedural sedation (Trapani et al., 2000). Its favorable pharmacokinetic profile (rapid onset and offset), excellent safety record, and well-characterized pharmacodynamics make it an attractive candidate for repurposing (Sahinovic et al., 2018). Several lines of evidence suggest that propofol may possess intrinsic antidepressant properties. First, propofol and ketamine, despite their distinct primary molecular targets (GABA_A_ receptors vs. NMDA receptors), share downstream effects on glutamatergic signaling and synaptic plasticity—mechanisms increasingly recognized as central to rapid antidepressant action (Duman et al., 2016). Second, recent controlled trials have begun to provide systematic evidence for propofol’s antidepressant efficacy in TRD. Finally, unlike ketamine, propofol is not a controlled substance with known abuse potential, potentially offering a more accessible and less regulated treatment option. This review provides a comprehensive synthesis of current knowledge regarding propofol therapeutics for psychiatric disorders, with an emphasis on depression.

Several recent reviews have summarized the antidepressant potential of anesthetic agents (Tadler and Mickey, 2018; Breault et al., 2025; Li et al., 2025). However, the present review differs from previous work in several important respects. First, we provide a propofol-focused synthesis rather than a broad overview of multiple agents. Second, we integrate the most recent clinical data from 2023–2025 (Feldman et al., 2025; Zeng et al., 2025). Third, we propose a three-stage integrative mechanistic model explaining how propofol’s brief action triggers sustained neuroadaptive changes (Zhu et al., 2023). Fourth, we systematically compare propofol with other anesthetic agents across multiple clinically relevant dimensions (Rhee et al., 2024; Breault et al., 2025; Mielko et al., 2025). Fifth, we highlight the recently discovered dopamine transporter inhibition as a non-GABAergic mechanism (Zhu et al., 2023). We address three main questions: (1) What clinical evidence supports propofol’s antidepressant effects? (2) What mechanisms mediate these effects? (3) How does propofol compare to other anesthetic agents being repurposed for psychiatric indications? We conclude by identifying key knowledge gaps and future directions for translating propofol from an experimental candidate to a clinically viable treatment.

We performed targeted searches in PubMed and Web of Science (inception to June 2026) using combinations of keywords including ‘propofol,’ ‘depression,’ ‘antidepressant,’ ‘treatment-resistant depression,’ and ‘anesthetic repurposing,’ supplemented by manual reference screening of key original studies and previous reviews. Given the narrative scope of this review, we selected literature based on relevance to the mechanistic, clinical, and comparative questions outlined below.

## 2 Clinical evidence for propofol’s antidepressant effects

### 2.1 Early observations and the TRD RCT

The possibility that propofol might influence mood emerged from clinical observations. Early case series reported transient mood elevation following propofol anesthesia. Retrospective analyses further suggested that patients receiving propofol-based anesthesia for electroconvulsive therapy (ECT) experienced less post-ictal confusion and faster recovery compared to other anesthetics (D'Haese et al., 1994; Harti et al., 2001; Walder et al., 2001; Bhakta et al., 2010). A study in healthy volunteers confirmed these observations, demonstrating that subanesthetic doses of propofol produce dose-dependent mood alterations, including euphoric and sedative effects (Zacny et al., 1992). Collectively, these early reports raised the possibility that propofol might possess intrinsic mood-modulating properties, although none were designed to systematically assess antidepressant efficacy.

Initial evidence from an open-label pilot study demonstrated that ten sessions of high-dose propofol targeting EEG burst suppression produced antidepressant responses in TRD patients (Mickey et al., 2018). In this proof-of-concept trial, repeated infusions to deep anesthetic levels led to clinically significant reductions in depressive symptoms, with some patients showing sustained improvement weeks after treatment. A subsequent randomized controlled trial has been conducted, and its secondary analysis on acute mood effects showed dose-independent improvements in sadness and fear, with no evidence of escalating abuse potential (Feldman et al., 2025). These results suggest that lower, safer doses may be therapeutically sufficient and that repeated propofol exposure does not appear to carry a significant risk of addiction in this clinical context.

### 2.2 Dose-dependence and acute mood effects

The dose-dependent nature of propofol’s antidepressant effect is a critical finding. Burst suppression, an electroencephalographic pattern indicating profound cortical inhibition, was associated with significantly greater mood improvement than sedation alone, suggesting that propofol’s antidepressant efficacy may require a threshold level of neurophysiological engagement (Mickey et al., 2018; Feldman et al., 2025). This pattern is conceptually analogous to the dose-response relationship observed with ketamine, where subanesthetic doses are effective while full anesthetic doses may not confer additional benefit (Shiroma et al., 2014; Sanacora and Schatzberg, 2015).

Secondary analyses have examined the acute effects of individual propofol infusions on mood dimensions. Propofol rapidly improves sadness, fear, happiness, and calmness. This acute mood elevation does not appear to be dose-dependent, as low and high doses produce comparable acute effects. However, with repeated infusions, the acute mood elevation diminishes, a pattern suggesting either tachyphylaxis or adaptive changes within mood-regulating circuits (Feldman et al., 2025). This finding, while preliminary, distinguishes propofol from ketamine and supports its favorable risk profile.

### 2.3 Propofol in electroconvulsive therapy

ECT is an established and highly effective treatment for severe depression, particularly in patients with treatment-resistant depression or those with psychotic features (Lisanby, 2007). Propofol has also been extensively studied as an anesthetic for ECT, where it is used to induce brief anesthesia prior to the convulsive stimulus. Compared to methohexital (a barbiturate historically preferred for ECT), propofol produces shorter seizure duration (Rasmussen, 2014). Since seizure duration was traditionally considered a marker of ECT efficacy, propofol was often avoided. However, contemporary evidence suggests that seizure duration is not a reliable predictor of antidepressant outcome; shorter seizures induced by propofol do not compromise ECT efficacy and may reduce cognitive side effects (Kales et al., 1997; Butterfield et al., 2004). The same systematic review noted that combining propofol with a short-acting opioid can prolong seizure duration, and concluded that all available induction agents are appropriate for ECT (Hooten et al., 2008). Several meta-analyses have compared propofol to other ECT anesthetics, concluding that propofol is associated with faster recovery, less post-ictal confusion, and perhaps lower rates of cognitive adverse effects compared to barbiturates (Huoponen et al., 2025). Evidence comparing etomidate and propofol for ECT induction is mixed; etomidate prolongs seizure duration but this metric poorly predicts clinical response, and etomidate carries risks of adrenocortical suppression and myoclonus. While direct antidepressant effects of propofol in ECT are confounded by the concurrent seizure, the favorable recovery profile and potential mood benefits warrant further investigation.

Recent comparative trials have extended this line of inquiry. A randomized, double-blind, non-inferiority trial directly compared esketamine versus propofol as anesthetic agents for ECT in patients with treatment-resistant depression (TRD) (Zeng et al., 2025). The study found that esketamine-ECT was non-inferior to propofol-ECT for reducing depressive symptoms after multiple sessions. Esketamine-ECT showed higher response and remission rates compared to propofol-ECT. In cognitive domains—including speed of processing, working memory, visual learning, and verbal learning—esketamine-ECT was non-inferior to propofol-ECT, suggesting that propofol-based ECT does not confer cognitive advantages over esketamine-based ECT (Zeng et al., 2025). Another ongoing randomized controlled trial protocol aims to compare propofol combined with different doses of esketamine versus propofol alone for ECT in patients with depression, with primary outcomes including HAMD and PHQ-9 scores (Chen X. et al., 2024). This trial may provide further insights into optimal combination strategies for ECT anesthesia (Chen X. et al., 2024).

### 2.4 Limitations of current evidence

Despite promising findings, the clinical evidence base for propofol’s antidepressant effects remains limited. Key limitations include small sample sizes, with no large-scale trials conducted to date. Follow-up duration is limited, leaving the durability of antidepressant effects unknown. There is a paucity of active comparator trials, although the recent esketamine comparison provides preliminary data (Zeng et al., 2025). Significant blinding challenges exist, as the profound sedation produced by high-dose propofol complicates blinded study designs, though the use of electroencephalographic burst suppression as a biomarker has helped standardize dosing. Generalizability remains uncertain, as all trials to date have focused on TRD, leaving efficacy unknown in non-resistant depression or other psychiatric conditions such as post-traumatic stress disorder, anxiety disorders, and bipolar depression (Feldman et al., 2025). A summary of key studies categorized by evidence type, population/model, main finding, and limitation is provided in **Table 1**.

**Table 1:** Summary of key studies on propofol by evidence type, population/model, main finding, and limitation.

Evidence type	Population/model	Main finding	Limitation	Ref.
Clinical trials				
Open-label pilot	TRD (n=10)	Antidepressant response; 5/6 responders well at 3 months	No control group; small sample size	(Mickey et al., 2018)
RCT secondary analysis	TRD (n=24)	Acute mood improvement; no escalating abuse potential	Not primary efficacy analysis	(Feldman et al., 2025)
ECT anesthesia studies				
Meta-analysis	Depression (multiple studies)	Propofol vs methohexital: equal ECT efficacy, faster recovery	Low-quality evidence; ECT confounds propofol effects	(Huoponen et al., 2025)
RCT (non-inferiority)	TRD (n=40)	Esketamine-ECT non-inferior to propofol-ECT	Propofol not administered alone	(Zeng et al., 2025)
Healthy volunteer studies				
Dose-response	Healthy (n=10)	Subanesthetic propofol produced dose-dependent mood alterations	Single dose; not depression population	(Zacny et al., 1992)
Animal models				
CUMS model	Mice	Single propofol reduced depression-like behavior; blocked by GABAA antagonist	Animal model; translation uncertain	(Song et al., 2026)
Chronic restraint stress	Mice	Propofol reversed anhedonia within 4h via DAT inhibition; effect lasted 3 days	Animal model; mechanism inferred from pharmacology	(Zhu et al., 2023)
Pain-anxiety model	Mice	Propofol alleviated anxiety via GABAA β3 subunits in PVN	Animal model; not depression-specific	(Yu et al., 2024)

## 3 Mechanisms of propofol’s putative antidepressant effects

The mechanisms discussed in this section derive primarily from preclinical studies in rodents, extrapolation from ketamine research, or studies of propofol in non-depressed populations (e.g., healthy volunteers, surgical patients). Direct evidence for propofol’s antidepressant mechanisms in humans with major depressive disorder or treatment-resistant depression is currently lacking.

### 3.1 GABAergic mechanisms: from receptor to circuit

Propofol is a positive allosteric modulator of γ-aminobutyric acid type A (GABA_A_) receptors. At clinically relevant concentrations, propofol binds to transmembrane β-subunit interfaces, increasing the receptor’s affinity for GABA and enhancing chloride ion flux (Trapani et al., 2000; Franks, 2008). Recent structural studies have identified three distinct classes of propofol binding sites on α1β3 GABA_A_ receptors, with evidence suggesting at least five propofol binding sites per pentamer (Chen ZW. et al., 2024). This allosteric enhancement produces hyperpolarization of postsynaptic neurons and reduced neuronal excitability—the basis for propofol’s sedative, hypnotic, and anesthetic effects (Malhi and Mann, 2018).

GABAergic dysfunction is increasingly recognized as a core component of depression pathophysiology (Duman et al., 2019). Postmortem studies have identified significant reductions in the density of calbindin-immunoreactive GABAergic neurons in the prefrontal and occipital cortices of depressed patients (Maciag et al., 2010). Consistent with these structural findings, the level of glutamic acid decarboxylase-67 (GAD-67), the GABA-synthesizing enzyme, is reduced by 34% in the dorsolateral prefrontal cortex in major depressive disorder (Karolewicz et al., 2010). Genetic association studies have linked GABA_A_ receptor subunit genes (particularly GABRA1, GABRA6, and GABRG2 located on chromosome 5q34) to mood disorders, with significant associations observed in female patients (Yamada et al., 2003; Barki and Xue, 2022). Importantly, some conventional antidepressants (e.g., SSRIs) increase cortical GABA levels after chronic administration (Ohira et al., 2013), and antidepressant medication appears to normalize GABA deficits in depression (Karolewicz et al., 2010). Beyond pharmacological interventions, repetitive transcranial magnetic stimulation (TMS), a non-invasive brain stimulation treatment for depression, has also been shown to elevate prefrontal cortex GABA levels in patients with major depressive disorder, further supporting the notion that normalization of GABAergic function is a convergent mechanism shared by diverse antidepressant treatments (Dubin et al., 2016).

Recent preclinical evidence directly supports propofol’s antidepressant-like effects through GABAergic mechanisms. Using a chronic unpredictable mild stress (CUMS) mouse model, Song and colleagues demonstrated that a single intraperitoneal injection of propofol (50 mg/kg) significantly reduced depression-like behaviors in both male and female mice (Song et al., 2026). Mechanistically, propofol increased medial prefrontal cortex (mPFC) GABAergic neuron excitability while suppressing glutamatergic activity, and restored CUMS-induced reductions in GABAergic and glutamatergic proteins. Pharmacologic inhibition of GABA_A_ receptors abolished propofol’s antidepressant effects, while optogenetic activation of mPFC GABAergic interneurons enhanced these effects (Song et al., 2026). Beyond depression, propofol also alleviates anxiety-like behaviors by inhibiting the hyperactivity of corticotropin-releasing hormone (CRH) neurons in the paraventricular nucleus (PVN) of the hypothalamus. This effect is mediated by GABA_A_ receptor β3 subunits, as knockdown of the β3 subunit in PVN^CRH^ neurons abolished propofol’s anxiolytic effect (Yu et al., 2024).

### 3.2 Dopamine transporter inhibition: a novel non-GABAergic mechanism

Propofol’s mood-elevating properties are not limited to GABAergic mechanisms. Using structural pharmacology, molecular docking, and behavioral assays, Zhu and colleagues discovered that propofol directly binds to and inhibits the dopamine transporter (DAT), but not the serotonin transporter (SERT) (Zhu et al., 2023). This inhibition leads to elevated dopamine levels specifically in the nucleus accumbens (NAc), a key brain region for reward processing. Fiber photometry recordings and [¹^8^F]FP-CIT-PET scanning confirmed that propofol administration evokes rapid and lasting dopamine accumulation in the NAc, driving biased activation of dopamine D1 receptor-expressing medium spiny neurons (D1-MSNs) (Zhu et al., 2023). In a chronic restraint stress model of depression, a single propofol injection reversed anhedonia-like behavior within 4 hours, an effect that persisted for up to 3 days. Chemogenetic inhibition of NAc D1-MSNs abolished this anti-anhedonic effect, demonstrating that propofol’s action on the dopaminergic system is both necessary and sufficient for its rapid anti-anhedonia effects (Zhu et al., 2023). In this study, antidepressant-like effects were assessed primarily through sucrose preference testing, a measure of anhedonia; the extent to which propofol affects other symptom domains of depression in this model remains to be fully characterized.

### 3.3 Convergence on glutamatergic plasticity and neurotrophic signaling

Despite their distinct primary mechanisms—GABA_A_ potentiation and DAT inhibition—propofol shares with ketamine a common downstream convergence on glutamatergic plasticity. Propofol-induced GABAergic enhancement may paradoxically enhance glutamatergic signaling through disinhibition: the release of downstream excitatory neurons from tonic GABAergic restraint. In depressive rats, propofol alleviates learning and memory impairment induced by electroconvulsive shock by regulating NMDA receptor-mediated metaplasticity, inhibiting NMDA receptor activation and lowering the long-term potentiation threshold (Kingston et al., 2006; Ren et al., 2018).

Brain-derived neurotrophic factor (BDNF) and its receptor TrkB are central to the neuroplasticity hypothesis of depression (Duman et al., 2016). Both ketamine and propofol increase BDNF signaling. In animal models, propofol administration modulates neuroplasticity pathways, likely mediated through activation of the mammalian target of rapamycin (mTOR) pathway, which promotes dendritic spine formation (Li et al., 2010). The balance between mature BDNF and its precursor proBDNF is critical for synaptic plasticity. Propofol improves cognitive function by downregulating the proBDNF/mBDNF ratio and increasing tPA expression, which converts proBDNF to mBDNF (Zhang et al., 2016). Propofol also selectively alters AMPA receptor trafficking. It increases phosphorylation of the GluA1 subunit at the Ser831 site, a modification associated with enhanced synaptic transmission. This effect is region-specific, occurring in the hippocampus but not the prefrontal cortex (Mao et al., 2014). Collectively, these findings demonstrate that propofol engages multiple glutamatergic plasticity mechanisms—including NMDA receptor metaplasticity, BDNF/proBDNF signaling, and AMPA receptor trafficking—that converge on promoting synaptic strengthening and may underlie its rapid antidepressant effects.

### 3.4 Network-level reorganization and an integrative model

Propofol profoundly reorganizes large-scale brain networks. At sedative doses, propofol reduces connectivity within the default mode network (DMN)—a set of brain regions more active during rest than during cognitive tasks (Guldenmund et al., 2017; Chen et al., 2022). DMN hyperconnectivity is a robust neuroimaging finding in depression, and normalization of DMN connectivity correlates with antidepressant response (Kaiser et al., 2015). Propofol also enhances slow-wave activity (0.5–4 Hz) in the EEG, a pattern associated with deep non-REM sleep (Murphy et al., 2011). Sleep disruption is both a symptom and a driver of depression, and interventions that restore normal slow-wave activity can transiently improve mood in depressed patients (Riemann et al., 2020). Additionally, propofol reduces cortisol secretion in stressed animals and humans (Sakai et al., 1995; Kurni et al., 2023), and hypercortisolemia with hypothalamic-pituitary-adrenal (HPA) axis dysregulation are well-established correlates of depression (Von Werne Baes et al., 2012; Machahary et al., 2025).

**Figure 1:**
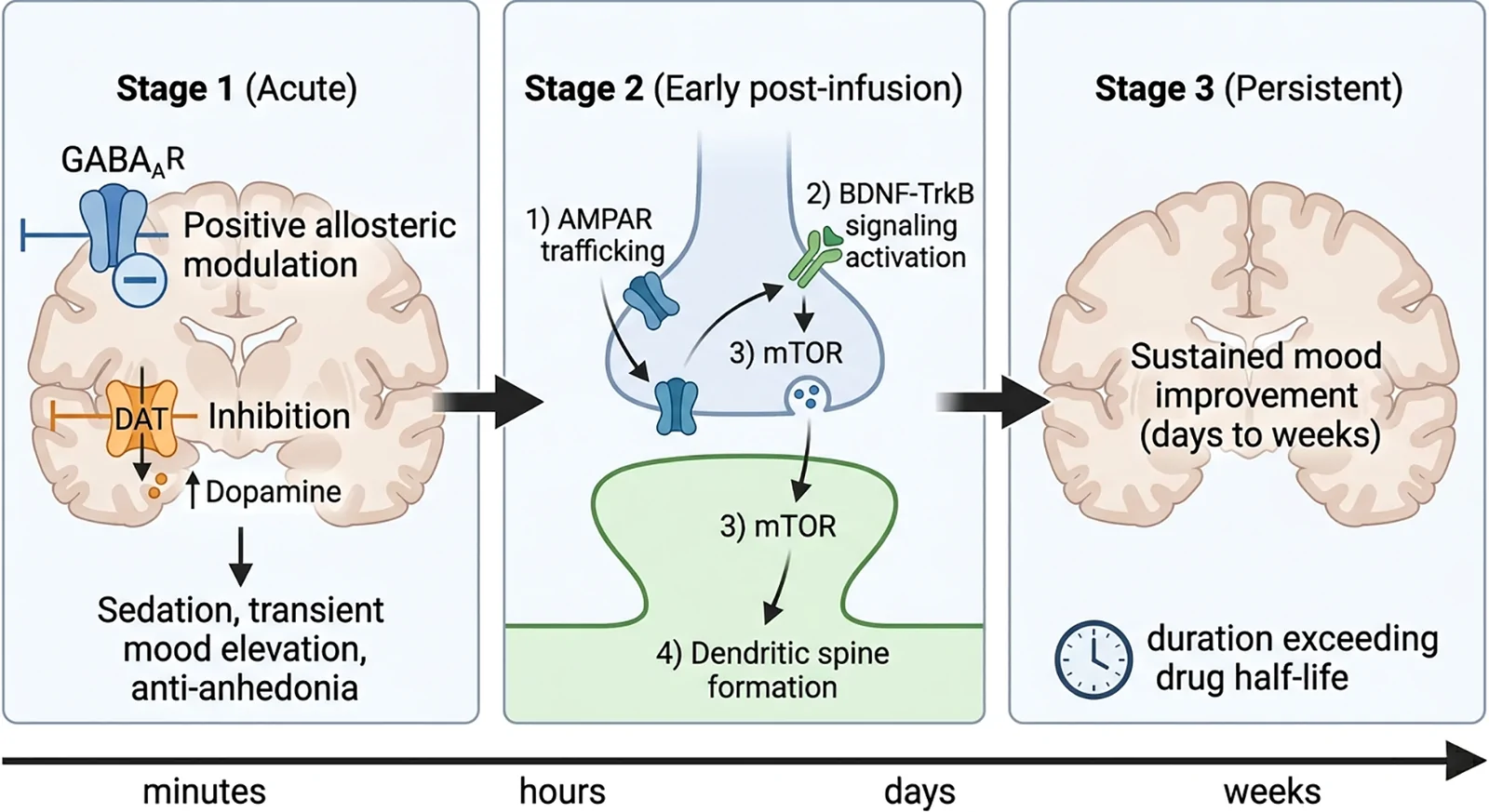
A proposed three-stage integrative hypothesis of propofol’s antidepressant mechanisms. This model is a conceptual hypothesis based on preclinical and indirect evidence. Direct demonstration of these mechanisms for propofol in depressed humans is lacking. The model is presented as a testable framework, not an established mechanism. In Stage 1 (Acute, minutes to hours), propofol acts as a positive allosteric modulator of GABAA receptors and inhibits the dopamine transporter (DAT), producing sedation, transient mood elevation, and anti-anhedonia effects. In Stage 2 (Early post-infusion, hours to 1 day), enhanced GABAergic inhibition and dopaminergic tone trigger compensatory homeostatic responses, including increased AMPAR trafficking and BDNF-TrkB-mTOR signaling, promoting dendritic spine formation in mood-regulating circuits. In Stage 3 (Persistent, days to weeks), synaptic structural changes stabilize, producing sustained mood improvement that outlasts propofol’s short half-life, positioning propofol as a “plasticity primer” whose brief primary pharmacologic effect triggers downstream neuroadaptations that endure beyond the drug’s presence in the body.

Synthesizing the evidence above, we propose an integrative multi-stage hypothesis that provides a testable framework (**Figure 1**). Stage 1 (Acute, minutes to hours): Propofol enhances GABA_A_ receptor function and inhibits DAT, producing sedation, transient mood elevation, and anti-anhedonia effects (Trapani et al., 2000; Zhu et al., 2023; Chen ZW. et al., 2024). Stage 2 (Early post-infusion, hours to 1 day): Enhanced GABAergic inhibition and dopaminergic tone trigger compensatory homeostatic responses, including increased AMPAR trafficking and BDNF-TrkB-mTOR signaling (Li et al., 2010). These plasticity events promote dendritic spine formation in mood-regulating circuits (prefrontal cortex, hippocampus, nucleus accumbens). Stage 3 (Persistent, days to weeks): Synaptic structural changes stabilize, producing sustained mood improvement despite propofol’s short half-life. This model posits that propofol acts as a “plasticity primer”—its brief primary pharmacologic effect triggers downstream neuroadaptations that outlast the drug’s presence in the body.

## 4 Propofol in the context of other anesthetic agents for depression

The success of ketamine has catalyzed systematic investigation into the antidepressant potential of multiple anesthetic agents (Breault et al., 2025). While ketamine remains the most extensively studied, accumulating evidence suggests that propofol, nitrous oxide, isoflurane, sevoflurane, and dexmedetomidine also possess intrinsic antidepressant properties, each with distinct mechanistic profiles and clinical considerations (Tadler and Mickey, 2018; Breault et al., 2025; Mielko et al., 2025; Zeng et al., 2025).

### 4.1 Ketamine: the prototype and benchmark

Ketamine, a non-competitive N-methyl-D-aspartate receptor (NMDAR) antagonist, represents the paradigm for rapid-acting antidepressants. Since the landmark report by Berman and colleagues in 2000, numerous clinical trials have confirmed that a single subanesthetic dose (0.5 mg/kg intravenously) produces rapid (within hours) and sustained (up to 7 days) antidepressant effects in patients with treatment-resistant depression (TRD) (Berman et al., 2000; Murrough et al., 2013; Krystal et al., 2019). The underlying mechanisms involve NMDAR blockade on GABAergic interneurons, leading to disinhibition of glutamatergic pyramidal neurons, increased extracellular glutamate, subsequent activation of α-amino-3-hydroxy-5-methyl-4-isoxazolepropionic acid receptors (AMPARs), and downstream signaling through brain-derived neurotrophic factor (BDNF) and mechanistic target of rapamycin (mTOR) pathways to promote dendritic spine synthesis (Duman et al., 2016; Krystal et al., 2019).

However, ketamine’s clinical utility is constrained by several limitations. Psychotomimetic effects (dissociation, perceptual disturbances) occur in a significant proportion of patients, raising tolerability concerns. The duration of antidepressant effect is transient, typically requiring repeated administration every 1–2 weeks. Long-term safety data remain limited, and concerns regarding bladder toxicity and cognitive impairment with chronic use have emerged. Additionally, ketamine is a Schedule III controlled substance with recognized abuse potential, restricting its accessibility (Breault et al., 2025).

### 4.2 Propofol vs. ketamine: distinct but convergent mechanisms

Before comparing propofol with ketamine, it is important to acknowledge that the clinical evidence base for propofol is substantially less established. Ketamine’s antidepressant efficacy is supported by dozens of RCTs, whereas propofol data are limited to one open-label pilot study (n=10) and one secondary analysis (n=24); moreover, direct comparisons have largely been conducted in the ECT setting, where propofol served as an anesthetic adjunct rather than an independent treatment (Zeng et al., 2025).

Propofol and ketamine differ fundamentally in their primary molecular pharmacology. Propofol is a positive allosteric modulator of GABA_A_ receptors, enhancing inhibitory neurotransmission, whereas ketamine is an NMDAR antagonist, primarily reducing glutamatergic excitation (Krystal et al., 2019). Despite these opposing actions, both agents converge on promoting glutamatergic plasticity—propofol through GABAergic disinhibition and ketamine through direct NMDAR blockade (Breault et al., 2025).

From a clinical perspective, a key advantage of propofol is that it does not produce psychotomimetic effects, making it more tolerable for patients in this regard. Ketamine, in contrast, commonly produces transient dissociation and perceptual disturbances. Propofol also has an exceptionally well-characterized safety profile accumulated over four decades of routine clinical use. However, propofol’s requirement for monitored anesthesia care and its dose-dependent respiratory depressant effects limit its accessibility compared to subanesthetic ketamine, which can be administered in outpatient psychiatric settings (Krystal et al., 2019; Zhu et al., 2023). Unlike ketamine, which is already a Schedule III controlled substance, propofol is not currently scheduled; however, its abuse potential is now recognized (see Section 5.2). A mechanistic and pharmacological comparison of propofol and ketamine is summarized in **Table 2**.

**Table 2:** Mechanistic and pharmacological comparison of propofol versus ketamine in the context of rapid antidepressant effects.

Feature	Propofol	Ketamine
Primary molecular target	GABA_A receptor positive allosteric modulator (Ruesch et al., 2012)	NMDAR non-competitive antagonist (Krystal et al., 1994)
Primary effect on neurotransmission	Enhances GABAergic inhibition	Reduces glutamatergic excitation via interneuron disinhibition
Downstream convergence	Glutamatergic plasticity (through GABAergic disinhibition) (Duman et al., 2016)	Glutamatergic plasticity (through direct NMDAR blockade) (Duman et al., 2016)
Typical dose for depression	High-dose to burst suppression (EEG-guided) (Mickey et al., 2018; Zhu et al., 2023)	Subanesthetic (0.5 mg/kg IV) (Berman et al., 2000)
Route of administration	Intravenous only	Intravenous, intranasal (esketamine)
Duration of effect after single dose	Unknown (requires repeated dosing)	Days to 1 week (Berman et al., 2000)
Common adverse effects	Respiratory depression, hypotension, sedation (Miner et al., 2010)	Dissociation, perceptual disturbances, hypertension, tachycardia (Krystal et al., 1994)
Psychotomimetic effects	None	Common (transient)
Abuse potential	Documented (tolerance, dependence, withdrawal, fatal overdoses). Recommended to be scheduled (Wilson et al., 2010; Uzbay and Shahzadi, 2025)	Moderate (Schedule III)
Regulatory status for depression	Investigational	FDA-approved (esketamine for TRD)
Administration setting	Operating room or ICU (monitored anesthesia care)	Outpatient clinic (with monitoring)
Safety database	Extensive (decades of clinical use in anesthesia)	More limited (psychiatric use since 2000)
Key advantage	No psychotomimetic effects	Rapid onset, FDA-approved, can be used in outpatient setting
Key limitation	Requires monitored anesthesia care, limited accessibility	Psychotomimetic effects, abuse potential, transient effect

### 4.3 Propofol in electroconvulsive therapy: comparative evidence

Propofol is one of the most commonly used anesthetics for ECT, where it provides rapid induction, ultra-short duration, and favorable recovery characteristics. However, propofol reduces seizure duration compared to barbiturates such as methohexital—a property that historically raised concerns about ECT efficacy.

Systematic reviews and meta-analyses have addressed this concern. A 2025 systematic review and meta-analysis comparing propofol and methohexital for ECT in depression found that the number of ECT sessions required for clinical remission did not differ significantly between groups (Huoponen et al., 2025). Across all included studies, depression scores significantly decreased from baseline in both treatment arms, indicating effective alleviation of depression regardless of anesthetic choice. The authors concluded that low-quality evidence suggests equal efficacy of propofol compared to methohexital with regard to clinical remission of depression after ECT (Huoponen et al., 2025). Importantly, propofol was associated with faster recovery, less post-ictal confusion, and improved hemodynamic stability compared to methohexital (Huoponen et al., 2025).

Network meta-analyses have further clarified the comparative profile of propofol. One analysis found that ketamine monotherapy ranked first in response and remission rates for ECT, but this benefit came with a higher incidence of adverse events. No significant advantage of any anesthetic was demonstrated for cognitive function after ECT (Ren et al., 2024). Another network meta-analysis specifically comparing ketamine-assisted ECT with other anesthetics confirmed that ketamine had better antidepressant efficacy than propofol when used as the ECT anesthetic. However, ketamine-treated patients showed worse cognitive outcomes compared to propofol-treated patients (Rhee et al., 2024). This cognitive advantage of propofol represents an important clinical consideration, particularly for patients vulnerable to ECT-related cognitive side effects (Zeng et al., 2025).

A randomized, double-blind, non-inferiority trial specifically compared esketamine, the S-enantiomer of ketamine, versus propofol as anesthetics for ECT in patients with TRD. Esketamine-ECT was found to be non-inferior to propofol-ECT for reducing depressive symptoms. In terms of cognitive function, including speed of processing, working memory, visual learning, and verbal learning, esketamine-ECT was also non-inferior to propofol-ECT. The authors concluded that esketamine represents an alternative anesthetic for patients with TRD undergoing ECT, while noting that replication studies with larger samples are needed (Zeng et al., 2025).

### 4.4 Other anesthetic agents with antidepressant potential

Nitrous Oxide. As an NMDAR antagonist, nitrous oxide has demonstrated rapid antidepressant effects in TRD. A proof-of-concept trial demonstrated that a single one-hour inhalation of 50% nitrous oxide produced significant antidepressant effects within hours in patients with TRD (Nagele et al., 2015; Gill et al., 2025). A subsequent phase 2 trial further established the efficacy and safety of nitrous oxide for treatment-resistant major depression (Nagele et al., 2021). Compared to ketamine, nitrous oxide produces fewer psychotomimetic effects and is not a controlled substance. It also possesses analgesic properties that may benefit patients with comorbid chronic pain. However, specialized administration equipment and concerns about environmental impact from greenhouse gas emissions limit its widespread adoption.

Isoflurane and Sevoflurane. These volatile anesthetics, like propofol, are positive allosteric modulators of GABAA receptors. Preclinical and clinical data suggest that isoflurane dosed to electroencephalographic burst suppression has relatively rapid, robust, and durable antidepressant effects, potentially offering an alternative to ECT that lacks adverse cognitive effects (Zhang et al., 2019; Li et al., 2025). However, clinical evidence remains mixed. A small double-blind randomized trial found that burst-suppression anesthesia (propofol induction followed by sevoflurane maintenance) did not significantly improve depression compared to sham anesthesia (Garcia-Toro et al., 2001). Environmental concerns also present sustainability challenges: sevoflurane and isoflurane have global warming potentials approximately 140 and 500 times that of carbon dioxide, respectively (Espinosa et al., 2025). Both agents also require specialized vaporizer equipment and scavenging systems, limiting accessibility compared to intravenous agents (Li et al., 2025).

Dexmedetomidine. This highly selective α2-adrenergic receptor agonist has shown antidepressant effects in preclinical models (Al-Alawi et al., 2020), with benefits in comorbid anxiety and neuropathic pain (Al-Mahrouqi et al., 2023). Clinical studies have confirmed its mood-modulating properties. In patients with chronic pain, intraoperative dexmedetomidine improves anxiety and depressive symptoms at one-month follow-up (Ren et al., 2025). Among older surgical patients, it reduces the incidence of postoperative depressive symptoms, anxiety, and sleep disturbance (Hao et al., 2025). In cesarean delivery, combining low-dose dexmedetomidine with esketamine lowers early postpartum depressive symptom scores and reduces neuropsychiatric adverse events compared to esketamine alone (Li et al., 2026). Dexmedetomidine can be administered intravenously or intranasally and produces minimal respiratory depression. However, its primary clinical effects are sedation and bradycardia, and efficacy may be limited to specific patient subgroups. Unlike propofol and ketamine, dexmedetomidine does not produce the seizure-inducing effects required for ECT. In modified electroconvulsive therapy, pretreatment with dexmedetomidine optimizes hemodynamic stability, reduces propofol requirements, and maintains seizure duration (Shen et al., 2025).

### 4.5 Comparative summary

Based on the available evidence, several key distinctions emerge when comparing propofol with other anesthetic agents for psychiatric indications. Ketamine demonstrates superior antidepressant efficacy, particularly in the ECT setting, but at the cost of more adverse effects including psychotomimetic symptoms and cognitive impairment (Anand et al., 2023; McGirr et al., 2015; Rhee et al., 2022; Movahed et al., 2023; Zeng et al., 2025). Propofol offers comparable efficacy to methohexital for ECT with better recovery profiles and potentially superior cognitive outcomes (Tadler and Mickey, 2018; Huoponen et al., 2025). The non-inferiority of esketamine to propofol as an ECT anesthetic provides clinicians with additional options, allowing patient-specific selection based on tolerability profiles (Zeng et al., 2025).

The clinical choice among these agents should be guided by individual patient characteristics, including the severity of treatment resistance, cognitive reserve, medical comorbidities, and tolerability of specific adverse effects. The expanding armamentarium of anesthetic-based therapies represents a paradigm shift from “one-size-fits-all” pharmacotherapy toward personalized, rapid-acting interventions for depression.

## 5 Challenges and future directions

Despite growing evidence supporting propofol’s antidepressant potential, the field faces substantial challenges across clinical, safety, and implementation domains. Addressing these issues will determine whether propofol transitions from an experimental candidate to a clinically viable treatment for depression.

### 5.1 Unresolved clinical questions

The dose-response relationship for propofol’s antidepressant effects remains imprecise. Available evidence suggests that burst suppression—an electroencephalographic pattern indicating profound cortical inhibition—may be necessary for maximal efficacy in treatment-resistant depression (TRD) (Mickey et al., 2018; Feldman et al., 2025). However, whether lower doses would suffice for less severely ill patients or for indications other than TRD is unknown. Dose-finding studies with systematic electroencephalographic monitoring are urgently needed to establish the minimum effective dose across different patient subgroups.

Pharmacological studies have established that the concentration required to induce burst suppression is substantially lower than that needed for surgical anesthesia, providing a benchmark for targeting this state in depression treatment (Pilge et al., 2014). Yet the diversity of burst patterns across individuals is considerable, indicating that fixed dosing protocols may not be optimal. A validated pharmacokinetic-pharmacodynamic model for high-dose propofol can improve prediction of burst-suppression ratio and support individualized dose optimization (Lybbert et al., 2023).

The optimal frequency and total number of propofol infusions have also not been established. The available pilot study used six infusions over approximately three weeks (Mickey et al., 2018), but whether more frequent or maintenance infusions would extend efficacy is unknown. A recent exploratory analysis of immediate mood effects from this trial found that propofol induced acute improvements in sadness, fear, joviality, and serenity at the first infusion, independent of dose; however, over the series of six infusions, acute changes in sadness, fear, and joviality diminished with infusion number (Feldman et al., 2025). This observation of diminishing acute mood effects with repeated dosing raises two possibilities: either tolerance develops to the mood-elevating effects, or the acute effects are not the primary driver of sustained antidepressant response. Distinguishing between these possibilities has important implications for treatment optimization.

Regarding durability, no studies have systematically followed patients beyond the immediate post-treatment period. The open-label pilot study reported that five of six responders remained well for at least three months (Mickey et al., 2018), but larger prospective studies with extended follow-up are needed to establish the duration of propofol’s antidepressant effects and determine whether maintenance infusions are necessary.

Electroencephalographic burst suppression is a candidate biomarker for target engagement, but its utility in predicting clinical response requires prospective validation. Studies have demonstrated that electroencephalographic measures predicted clinical response to propofol in treatment-resistant depression, and specific burst patterns have been proposed as electrical biomarkers correlated with patient outcomes (Lybbert et al., 2023). Other candidate biomarkers—including prefrontal cortical brain-derived neurotrophic factor levels, neuroimaging measures of default mode network connectivity, and peripheral inflammatory markers—remain exploratory at present. Head-to-head trials comparing propofol to ketamine, electroconvulsive therapy (ECT), and conventional antidepressants are needed to position propofol within the treatment algorithm for depression. Such trials should include both efficacy outcomes (depression severity, response, remission) and tolerability outcomes (cognitive function, adverse events, quality of life).

### 5.2 Safety and tolerability

Propofol produces dose-dependent respiratory depression and hypotension, requiring administration by trained personnel in settings equipped for airway management (Sahinovic et al., 2018; Green and Krauss, 2008). Unlike ketamine, which can be administered in outpatient settings with appropriate monitoring, propofol infusions to anesthetic depths (burst suppression) typically require an operating room or intensive care setting. This requirement limits accessibility and substantially increases cost.

The safety of repeated propofol exposures has been demonstrated in a small randomized controlled trial using six infusions over three weeks (Mickey et al., 2018). However, the safety of more frequent or prolonged courses remains unknown. Propofol infusion syndrome (PRIS) is a rare but potentially fatal complication characterized by metabolic acidosis, rhabdomyolysis, cardiac failure, and renal failure (Krajcova et al., 2015; Hemphill et al., 2019). PRIS was first identified in critically ill children receiving prolonged high-dose propofol infusions and has since been reported in adults, primarily when propofol is administered at high doses for extended periods (Vasile et al., 2003; Fong et al., 2008). The pathophysiology involves propofol-induced impairment of mitochondrial fatty acid oxidation and disruption of the electron transport chain, leading to cellular energy failure (Karasawa et al., 2020).

Notably, case series have documented that PRIS can occur even at infusion rates below traditional safety thresholds, with cardiac failure and metabolic acidosis emerging early in a dose-dependent manner (Krajcova et al., 2015). The incidence of PRIS in critically ill adult populations is approximately 3%, with an associated mortality rate exceeding one-third (Li et al., 2022). In patients receiving intermittent propofol for depression, the risk of PRIS appears low given the short duration and intermittent nature of the exposures. However, screening protocols measuring creatine phosphokinase and lactate levels have been proposed for early detection in at-risk populations (Schroeppel et al., 2014). Given the high mortality associated with PRIS, this risk, although rare, warrants careful consideration when repeated propofol infusions are contemplated.

Contrary to earlier assumptions, propofol is now recognized to have abuse liability. Multiple case series and systematic reviews have documented tolerance, dependence, withdrawal symptoms, and fatal overdoses associated with propofol misuse, leading some authors to propose its classification as a controlled substance (Wilson et al., 2010; Uzbay and Shahzadi, 2025). Notably, dependence has also been reported in patients with no history of substance abuse, acquired solely through clinical anesthetic exposure (Koopmann et al., 2011). Mechanistically, propofol inhibits the dopamine transporter in the nucleus accumbens, a property shared with known drugs of abuse such as cocaine, providing a neurobiological basis for its rewarding effects (Zhu et al., 2023). Collectively, these findings indicate that propofol’s abuse liability is a significant safety concern that must be carefully weighed against its therapeutic potential, particularly for repeated outpatient administration.

Propofol’s cognitive effects at subanesthetic doses are not well characterized. Unlike ketamine, which can produce transient cognitive impairment, propofol’s effects on cognition may resolve rapidly due to its short half-life (Tadler and Mickey, 2018). However, formal neuropsychological testing before and after propofol treatment courses has not been reported. Given that many patients with depression already experience cognitive dysfunction, this represents an important knowledge gap. No long-term safety data exist for repeated propofol exposures in psychiatric populations. Long-term adverse effects of concern include potential neurotoxicity—particularly with repeated high-dose exposures—hepatic dysfunction (propofol is metabolized in the liver), and effects on sleep architecture (Sahinovic et al., 2018; Lu et al., 2026; Yue et al., 2021).

### 5.3 Implementation barriers

Propofol is off-patent, which substantially reduces commercial incentive for pharmaceutical companies to pursue regulatory approval for a new indication. Without industry sponsorship, large-scale registration trials will be difficult to fund. Academic consortia, government funding, or non-profit foundations may need to shoulder this responsibility.

Propofol’s requirement for monitored anesthesia care—typically an anesthesia provider present throughout the infusion—is a major barrier to widespread adoption (Green and Krauss, 2008). If propofol is ultimately approved for TRD, development of simpler administration protocols might expand access, though any such protocols must prioritize patient safety. Off-label propofol for depression is not currently reimbursed by most insurers, causing patients to pay out-of-pocket and creating access disparities. Establishing a reimbursement pathway will require a combination of regulatory approval and practice guideline development.

### 5.4 Future research priorities

Based on the current evidence gaps, priority areas for future research include: (1) large-scale randomized controlled trials comparing propofol to sham sedation or active comparators (ketamine, ECT, conventional antidepressants); (2) mechanistic studies in humans using neuroimaging and neurophysiology to map neural correlates of propofol-induced mood improvement; (3) prospective validation of candidate biomarkers including electroencephalographic burst suppression; (4) systematic dose-finding studies to identify minimum effective doses for different patient subgroups; (5) pilot studies exploring propofol’s efficacy in bipolar depression and peripartum depression; (6) long-term safety registries to assess risks of repeated exposures; and (7) comparative effectiveness trials against established treatments.

## 6 Conclusion

Propofol represents a promising candidate for the emerging field of anesthetic repurposing in psychiatry. Preliminary clinical evidence, though extremely limited, suggests that propofol may produce dose-dependent antidepressant effects in treatment-resistant depression, with an acute safety profile that is well-characterized from decades of surgical use. However, propofol is now recognized to have abuse liability (Wilson et al., 2010; Uzbay and Shahzadi, 2025), a safety concern that must be carefully considered in any repeated or outpatient psychiatric application. Mechanistic studies suggest convergence of propofol and ketamine on glutamatergic plasticity and synaptic potentiation, despite their distinct primary molecular targets. Propofol’s advantages include its lack of psychotomimetic effects and controlled substance status, whereas its requirement for monitored anesthesia care presents implementation challenges.

The path to clinical adoption requires large-scale trials, biomarker development, and solutions to implementation barriers. From a mechanistic perspective, propofol offers a distinct approach that converges on shared downstream plasticity pathways with ketamine, warranting further clinical investigation. However, its clinical evidence base remains preliminary, and propofol should not be positioned as a clinical alternative to ketamine until prospective randomized trials with primary efficacy endpoints are completed. For the millions of patients suffering from treatment-resistant depression, expanding the armamentarium of rapid-acting treatments remains an urgent clinical priority. Propofol therapeutics, while still in early stages, merits continued investigation as part of this broader effort.

The convergence of anesthesiology and psychiatry—exemplified by propofol’s repurposing—represents a new frontier in neuropsychiatric therapeutics. As mechanistic understanding deepens and clinical evidence accumulates, propofol may transition from an operating room essential to a valuable tool in the treatment of debilitating psychiatric disorders.
